# Current perspectives in obesity management: unraveling the impact of different therapy approach in real life obesity care

**DOI:** 10.1186/s12967-024-05322-4

**Published:** 2024-06-06

**Authors:** Keyvan Khorrami Chokami, Amir Khorrami Chokami, Giuseppe Cammarata, Grazia Piras, Manuela Albertelli, Federico Gatto, Lara Vera, Diego Ferone, Mara Boschetti

**Affiliations:** 1 Endocrinology Unit, Department of Internal Medicine and Medical Specialties (DiMI), University of Genova, Genoa, 16132 Italy; 2 ESOMAS Department, University of Turin and Collegio Carlo Alberto, Turin, Italy; 3 Endocrinology Unit, IRCCS Ospedale Policlinico San Martino, Genoa, 16132 Italy

**Keywords:** Weight loss interventions, Metabolic parameters, Lipid profile, Anti-obesity medications, Ketogenic diet, Glycemic profile

## Abstract

**Background:**

The challenge of addressing obesity persists in healthcare, necessitating nuanced approaches and personalized strategies. This study aims to evaluate the effects of diverse therapeutic interventions on anthropometric and biochemical parameters in individuals with overweight and obesity within a real-world clinical context.

**Methods:**

A retrospective analysis was conducted on 192 patients (141 females, 51 males) aged 18 to 75, with a BMI ranging from 25 to 30 (14.1%) and BMI ≥ 30 (85.9%), observed over a 12-month period at our Endocrinology Unit. Treatment cohorts comprised individuals following different regimens: Mediterranean Diet (MD), with an approximate daily intake of 1500 kcal for women and 1800 kcal for men (71% patients); Ketogenic Diet (KD), utilizing the VLCKD protocol characterized by a highly hypocaloric dietary regimen < 800 kcal/day (14% patients); metformin, administered using the oral formulation (5% patients); pharmacological intervention with GLP1-RA administered via subcutaneous injection with incremental dosage (10% patients). Supply constraints limited the efficacy of Liraglutide, whereas Semaglutide was excluded from comparisons due to its unavailability for obesity without diabetes. Blood tests were conducted to assess lipid profile, glycemic profile, and anthropometric parameters, including BMI, waist circumference, and waist-to-height ratio.

**Results:**

Significant BMI changes were observed from baseline to 6 months across MD, KD, and Liraglutide groups (*p* < 0.05). KD exhibited notable reductions in waist circumference and waist-to-height ratio within the initial quarter (*p* < 0.05), with a significant triglyceride decrease after 6 months (*p* < 0.05), indicating its efficacy over MD. Liraglutide demonstrated a substantial reduction in HbA1_c_ levels in the first quarter (*p* < 0.05). During the first three months, the ANOVA test on fasting blood glucose showed a statistically significant impact of the time variable (*p* < 0.05) rather than the specific treatments themselves (Liraglutide and KD), suggesting that adherence during the early stages of therapy may be more critical than treatment choice.

**Conclusions:**

Positive outcomes from targeted interventions, whether pharmacological or dietary should encourage the exploration of innovative, long-term strategies that include personalized treatment alternation. The absence of standardized protocols underscores the importance of careful and tailored planning in managing obesity as a chronic condition.

## Introduction

The therapeutic management of obesity presents an ever-evolving challenge for healthcare professionals dedicated to addressing this condition. Describing obesity as a chronic, relapsing, and progressive condition [1], it becomes imperative to delve into the intricate network that underlies its origin and progression [2]. As well known, we can classify obesity using a widely used tool in clinical practice, the Body Mass Index (BMI). This index essentially involves the ratio of body weight to height squared (kg/m^2^), with obesity conventionally defined as a BMI ≥ 30 (kg/m^2^) [3].

Another easily applicable tool in clinical practice, especially for assessing visceral adipose tissue in the abdominal region, is waist circumference. The threshold values for increased cardio-metabolic risk are greater than 88 cm for women and greater than 102 cm for men [3]. Abdominal obesity is known to be associated with an increased cardiovascular risk and serves as a fundamental diagnostic criterion for the diagnosis of metabolic syndrome. The excess of abdominal visceral adipose tissue is the predominant manifestation of metabolic syndrome [4]. It can be utilized as a marker of “dysfunctional adipose tissue” and holds crucial importance, but improved risk assessment algorithms are needed to quantify the risk associated with adipose tissue-related diseases [5]. In this study, we also underscore the significance of the waist-to-height ratio, a simple and practical index for evaluating central fat distribution and metabolic risk. By assessing it in conjunction with the widely recognized waist circumference, more comprehensive analyses can be carried out, identifying a stratified age-specific cutoff of 0.5–0.6 [6].

Effective management in this context holds significant importance in averting atherosclerosis, cardiac remodeling, and the ensuing onset of ischemic heart disease and heart failure—acknowledged mortality factors among individuals with obesity [7]. Furthermore, chronic inflammation of adipose tissue also constitutes a substantial contributing factor to increased mortality. An example of this relationship was the recent Covid-19 pandemic, where obesity exacerbated low-grade systemic inflammation, leading to higher hospitalization rates and increased disease severity. This highlighted how obesity-related chronic inflammation can compromise the immune response, thereby accentuating the negative consequences of Covid-19 [8].

Considering the complexity of obesity, a multidisciplinary approach is necessary to establish proper therapeutic management. Patient phenotyping holds significance and entails an analysis encompassing the distribution/types of body fat, risk factors, associated pathologies, and subjective symptomatology. Regarding the investigation into the subjective symptoms of patients with obesity, the Edmonton Obesity Staging System (EOSS) is a staging system based on the clinical symptomatology reported by the patient and its impact on daily life [9]. Considering the therapeutic management, it is firmly established that pharmacotherapy for obesity represents a fundamental tool for proper disease management but cannot be separated from adequate lifestyle modification. Among the long-approved drugs for obesity treatment are certainly GLP1-RA, particularly liraglutide and semaglutide. Glucagon-Like Peptide-1 Receptor Agonists (GLP1-RA) enhance glucose-dependent insulin secretion while concurrently diminishing glucagon production and delaying gastric emptying. At the same time, these agents hinder food intake and promote weight reduction [10]. They now represent a cornerstone in the therapeutic approach to obesity. Liraglutide allows for once-daily administration [11] while semaglutide differs in that only weekly administration is required [12]. Although not yet available in many countries, since the years 2021 and 2022, it has been approved in both the United States and Europe by the respective regulatory agencies (FDA and EMA) for the treatment of overweight complicated by comorbidities and obesity, with a dosage of 2.4 mg per week [13, 14]. Present pharmaceutical intervention is advised for individuals with a BMI ≥ 30 (kg/m^2^) or a BMI ≥ 27 (kg/m^2^) coupled with a disease linked to obesity [15, 16]. Equally well-known is the paradigm of dietary therapy: for decades, the benefits of the Mediterranean Diet (MD) have been documented in literature, and, more recently, the advantages and indications of the Ketogenic Diet (KD) for obesity therapy have become firmly established. The dietary approach must be tailored, considering the specific characteristics of the patient, any existing comorbidities, the level of physical activity, body weight and dietary history, as well as past efforts aimed at weight reduction [15].

The Ketogenic Diet (KD), originating in the 1920s as a dietary therapeutic regimen for refractory epilepsy in children, involves a pronounced and evident restriction of carbohydrates [17]. It has showcased promising implications in cancer, as well as protective functions for the heart and liver. This, in turn, has ushered in fresh therapeutic perspectives for conditions associated with obesity and cardiovascular diseases [18, 19]. The acronym VLCKD refers to a “Very Low-Calorie Ketogenic Diet,” a highly hypocaloric dietary regimen (< 800 kcal/day) characterized by minimal daily carbohydrate intake, less than 30–50 g/day [20, 21]. The extensively documented advantages linked to VLCKD in addressing body weight and metabolic parameters in individuals with obesity include substantial decreases in body weight and body mass index (BMI), a notable reduction in waist circumference and overall fat mass, decreased levels of blood glucose and glycated hemoglobin (HbA1_c_), a decline in the HOMA index [22], heightened insulin sensitivity, and ultimately, a notable decrease in total cholesterol—exhibiting a more pronounced impact when compared to alternative weight-loss diets [21]. The therapeutic protocol for the KD includes three distinct phases: Active Stage, Re-education Stage, and Maintenance Stage. Subsequent to the reintegration phase, the regimen progresses to a Maintenance Stage (with caloric intake ranging from 1500 to 2000 kcal/day), characterized by tailored refinements, strategically crafted to perpetuate enduring weight loss [20, 21].

In this context, our study aims to address the need for specialized obesity centers to synergistically integrate dietary and pharmacological treatments, leveraging their distinct therapeutic effects. The underlying hypothesis is to demonstrate how various treatments, administered at specific intervals, influence anthropometric and metabolic parameters in patients undergoing treatment at our dedicated center. The primary objective of this retrospective cohort study is to conduct comprehensive analyses and comparisons on the effectiveness of various therapeutic strategies for managing obesity in a real-world context, focusing on their impact on anthropometric and hematological parameters commonly evaluated in clinical practice, in relation to the various proposed interventions.

## Methods

The cross-sectional observational study comprised a patient cohort of 192 adult patients, consisting of 141 females (73%) and 51 males (27%), all with overweight or obesity [with a BMI (kg/m^2^) ranging from 25 to 29.9 (14.1%) and BMI ≥ 30 (85.9%)], over an overall follow-up period of 12 months. Participants were enrolled at the Department of Internal Medicine and Medical Specialties, Endocrinology Unit, “Policlinico San Martino” Hospital, University of Genoa. The data collected during the study were recorded in an electronic medical system and then transferred into a comprehensive database after obtaining signed informed consent. The study followed the guidelines specified in the Declaration of Helsinki, and the Local Ethical Committee approved the study protocols (Protocol Project Number 377/2023 DB id 13324). All participants were required to provide written informed consent. After obtaining consent, 287 participants were enrolled consecutively. The inclusion criterion was to select patients with overweight or obesity, followed at the Endocrinology Unit, aged between 18 and 75 years old.

Conversely, the following criteria were employed for exclusion:

- Unstable medical conditions: patients with severe or unstable medical conditions, such as severe heart diseases, advanced renal failure, uncontrolled diabetes, or advanced oncological disorders.
- Severe psychiatric disorders: Patients with severe psychiatric disorders that could interfere with therapeutic adherence.
- Pregnancy or breastfeeding.
- Specific endocrine conditions: Patients with specific pathologies associated with active secondary causes of obesity.
- Use of interfering medications affecting weight loss: Individuals taking medications known to significantly influence body weight without indication for obesity.

Based on the indicated exclusion criteria, 192 participants were included in the analysis. For each patient, a thorough medical history, personal and family history, vital signs, physical examination, dietary history, dietitian consultation, and, finally, bioelectrical impedance analysis (BIA) were collected. We divided the patients into 4 groups based on the dietary treatment (Mediterranean diet or Ketogenic diet) or therapeutic treatment (liraglutide, semaglutide, or metformin). Medical treatment was always associated with the Mediterranean diet. The original pharmacological treatments that were already administered to the patients before the treatments proposed in the study (interfering with the parameters analyzed) were not modified during the duration of study. Regarding patients taking semaglutide, they were subsequently excluded from the evaluation of metabolic parameters because this drug was administered for the presence of diabetes and not for the therapeutic indication of obesity.

Therefore, the therapeutic groups were as follows:

- Group 1: Mediterranean Diet (MD), a hypocaloric approach based on the Mediterranean Diet model, personalized and tailored to each patient (91 F; 43 M; average age F/M 52/54 years);

In our study, treatments involving a Mediterranean hypocaloric diet were structured to limit average energy intake to approximately 1500 kcal per day for women and 1800 kcal per day for men, with fat constituting no more than 35% of total caloric intake. The primary sources of added fats comprised 25–35 g of olive oil, and all patients were encouraged to incorporate the consumption of nuts into their diets [23];

- Group 2: Pharmacological therapy with GLP1-RA involves the use of medications like liraglutide, administered daily via subcutaneous injection with incremental dosage (21 F; 5 M; average age of F/M 49/57 years); the dosage most frequently prescribed was 1.8 mg/day. The remaining patients in therapy with GLP1-RA were excluded from the analysis because they were undergoing treatment with semaglutide, which was unavailable for treating obesity without diabetes at the time of the study.
- Group 3: Ketogenic Diet (KD), a protocol involving a 4-week period of “Very Low-Calorie Ketogenic Diet” (VLCKD) followed by a gradual reintroduction of carbohydrates for an additional 4 weeks, then transitioning to a maintenance phase with a personalized MD (6 F; 3 M; average age F/M 40/45 years). The VLCKD protocol we utilized is characterized, as previously mentioned, by a highly hypocaloric dietary regimen (< 800 kcal/day), with the following macronutrient composition: minimal daily carbohydrate intake, less than 30–50 g/day (approximately 13% of total energy intake); protein intake of 1–1.5 g/kg of ideal body weight (approximately 43%); fat intake of 15–30 g/day (approximately 44%) [20, 21].
- Group 4: Metformin Therapy: administered to patients with obesity and insulin resistance using the traditional oral formulation (19 F; average age 43 years); dosage prescribed 500–1500 mg/day.

The table (Table 1) shown below presents all the baseline study cohort information.

**Table 1:** Clinical data for study cohorts at baseline, categorized by gender and treatment group

Gender	Group	Patients (%)	Age (years)	BMI (kg/m^2^)	Tot_Chol (mg/dL)	LDL (mg/dL)	TG (mg/dL)	Crfwaist (cm)	CrfHratio	FG (mg/dL)	HbA1c (%)	HOMA-IR
F	1	48.66	51.8 ± 5.42	36.37 ± 2.69	197.12 ± 7.5	121.67 ± 17.05	117.84 ± 23.02	111.23 ± 3.19	0.69 ± 0.02	98.26 ± 4.53	5.77 ± 0.07	5.52 ± 1.08
F	2	11.23	48.81 ± 5.42	37.67 ± 2.69	185.28 ± 7.5	116.98 ± 17.05	103.35 ± 23.02	114.88 ± 3.19	0.72 ± 0.02	94.21 ± 4.53	5.65 ± 0.07	6.69 ± 1.08
F	3	3.21	39.83 ± 5.42	42.3 ± 2.69	203.33 ± 7.5	153.5 ± 17.05	139.33 ± 23.02	119 ± 3.19	0.73 ± 0.02	88.25 ± 4.53	5.6 ± 0.07	6.23 ± 1.08
F	4	10.16	43.05 ± 5.42	37.03 ± 2.69	196.06 ± 7.5	120.21 ± 17.05	155.47 ± 23.02	115.63 ± 3.19	0.71 ± 0.02	89.76 ± 4.53	5.64 ± 0.07	4.21 ± 1.08
M	1	22.46	53.43 ± 6	37 ± 1.6	201.59 ± 15.79	132.05 ± 15.32	131.27 ± 20.92	121.83 ± 7.79	0.7 ± 0.03	95.83 ± 6.69	5.63 ± 0.21	7.8 ± 10.53
M	2	2.67	56.6 ± 6	39.92 ± 1.6	175.25 ± 15.79	110.25 ± 15.32	110.5 ± 20.92	136.4 ± 7.79	0.74 ± 0.03	106.2 ± 6.69	5.45 ± 0.21	7.54 ± 10.53
M	3	1.6	45 ± 6	39.59 ± 1.6	173.33 ± 15.79	102.5 ± 15.32	152.33 ± 20.92	124.33 ± 7.79	0.68 ± 0.03	108.33 ± 6.69	5.87 ± 0.21	25.9 ± 10.53

Data are expressed as mean ± SD

*Group 1* Mediterranean Diet (MD); *Group 2* Liraglutide; *Group 3* Ketogenic Diet (KD); *Group 4* Metformin

*BMI* body mass index, *LDL* low-density lipoprotein, *TG* triglycerides, *Crfwaist* waist circumference, *CfrHratio* waist-to-height ratio, *FG* fasting glucose, *HbA1*_*c*_ Glycated Hemoglobin, *HOMA-IR* Homeostatic Model Assessment of Insulin Resistance

For each patient, blood tests were conducted concerning the lipid profile (total cholesterol, triglycerides, LDL), glycemic profile (fasting blood glucose, HbA1_c_, HOMA-IR), and anthropometric parameters were collected, including BMI, waist circumference, and waist-to-height ratio. Blood samples were collected in the early morning, after an overnight fast, for the analysis of all the above-mentioned parameters that were analyzed by automatic routine methods in use at the Medicine Laboratory of our Institution (“Policlinico San Martino”, Genoa, Italy). LDL cholesterol concentrations were calculated using the well-known Friedewald formula [24].

Treatment groups, whether receiving medication or undergoing the Ketogenic Diet, adhered to the provided guidelines, systematically excluding cases with contraindications. A specific variable and the appropriate follow-up time/therapy group were selected. This approach complements graphical representations with more pertinent statistical data. In consideration of this, graphs were generated, depicted using violin plots and density curves, and accompanied at the bottom by box plots, offering a more comprehensive view of the trends and medians for each therapy group. The value indicated on the y-axis at each point is a measure of how likely it is that a generic measurement falls extremely close to the corresponding point on the x-axis [25]. The graphical representation starts with an analysis of BMI, followed by the assessment of waist circumference, and subsequently, an examination of the lipid profile (triglycerides, total cholesterol, and LDL), and the glycemic profile (fasting blood glucose, HbA1_c_, HOMA-IR).

**Map. 1:**
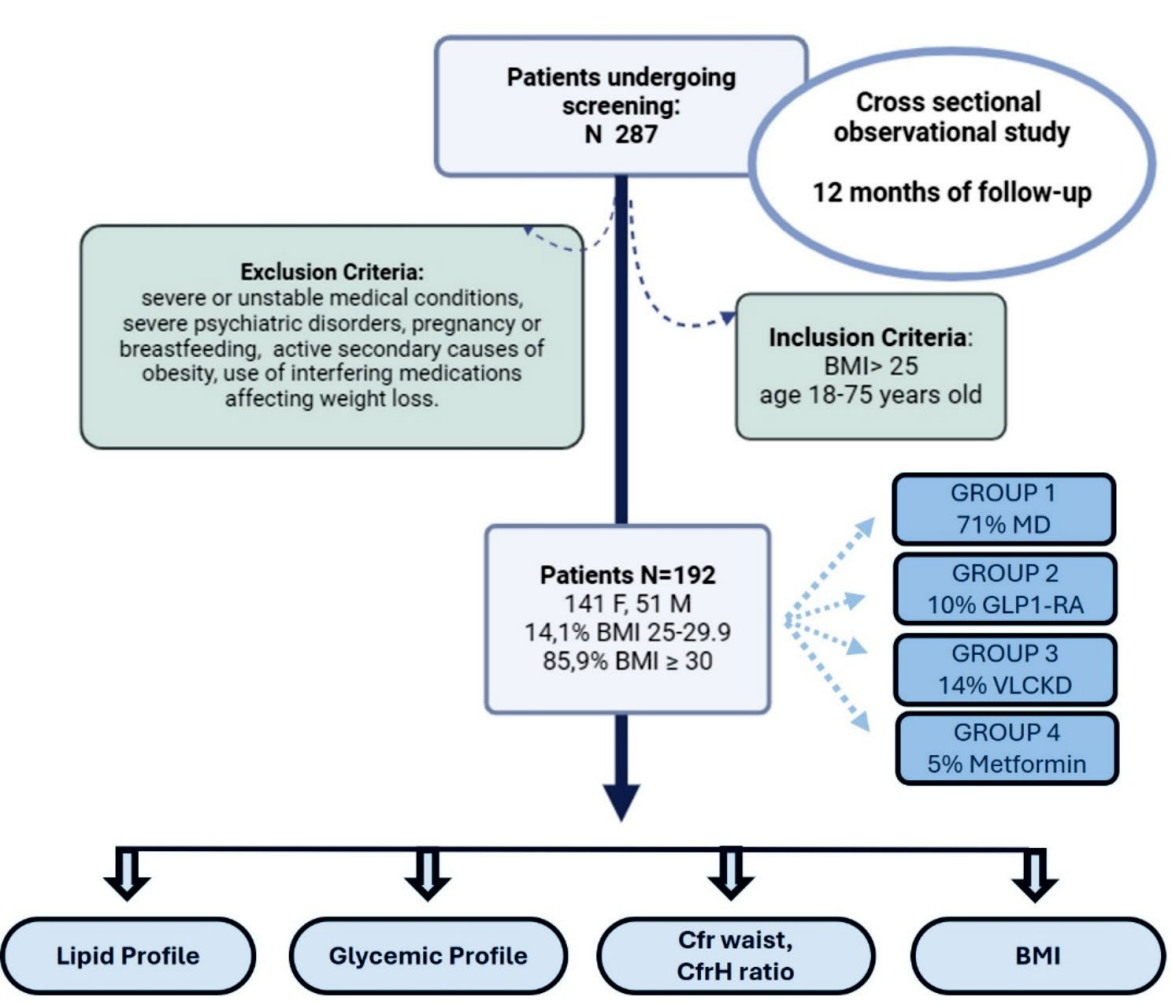
Map illustrating the clinical design of the study

The clinical design of the study is illustrated in the “Study Map” (Map 1).

### Statistical analysis

For the analysis and creation of graphs, as well as the execution of statistical tests, R software (version 4.2.2) was employed. Various types of graphical representations, including density curves, box plots, and violin plots, were generated to achieve a comprehensive and accurate analysis of the data. To examine statistically significant differences among therapy groups over time, Analysis of Variance (ANOVA) was used [26]. Comparisons between variables at different follow-up times were conducted using paired t-tests [p-values are below 0.001 (***), between 0.001 and 0.01 (**) and between 0.01 and 0.05 (*)]. Specific sub-analyses were focused on a maximum period of 12 months. This approach facilitated a more accurate presentation of data and relationships within the study.

## Results

The descriptive analysis of the results concerning the 192 adult patients, composed of 141 females (73%) and 51 males (27%), resulted in a trend comparison of anthropometric variables and laboratory parameters. The following table (Table 2) aims to display the mean values and standard deviations (SD) of the variables analyzed at baseline and at various follow-up times at 3, 6, and 12 months.

Changes in mean values of various parameters for each therapy group utilized can be observed.

**Table 2:** Mean values and standard deviations (SD) of the analyzed variables

Time (months)	Group	BMI (kg/m^2^)	Tot. Chol. (mg/dL)	LDL (mg/dL)	TG (mg/dL)	Crfwaist (cm)	CrfHratio	FG (mg/dL)	HbA1_c_ (%)	HOMA-IR
0	1	36.6 (± 6.5)	197.7 (± 38.7)	124.1 (± 36.5)	122.3 (± 66.3)	114.6 (± 15.2)	0.7 (± 0.1)	97.5 (± 19.4)	5.8 (± 0.7)	6.2 (± 8.4)
0	2	38.1 (± 6)	183.5 (± 32.6)	115.3 (± 31.5)	104.7 (± 37.3)	120 (± 16)	0.7 (± 0.1)	96.7 (± 14.6)	5.6 (± 0.4)	6.8 (± 8.1)
0	3	41.4 (± 5.5)	188.3 (± 41.6)	128 (± 39.7)	145.8 (± 22.8)	120.8 (± 11.3)	0.7 (± 0.1)	96.9 (± 13.7)	5.7 (± 0.5)	11.2 (± 10.7)
0	4	37 (± 6.9)	196.1 (± 29.6)	120.2 (± 30)	155.5 (± 85.8)	115.6 (± 11.8)	0.7 (± 0.1)	89.8 (± 12.4)	5.6 (± 0.5)	4.2 (± 3.2)
3	1	36.7 (± 6.8)	186.5 (± 38.3)	108.1 (± 31)	113.6 (± 45.6)	116.4 (± 17.3)	0.7 (± 0.1)	92.8 (± 12.1)	5.7 (± 0.3)	5.2 (± 6.3)
3	2	36 (± 6)	184.2 (± 40.8)	91.9 (± 27.5)	118.1 (± 40.9)	115.5 (± 17.3)	0.7 (± 0.1)	89.3 (± 8.1)	5.4 (± 0.3)	6.4 (± 9.4)
3	3	37.2 (± 5.8)	158.6 (± 43.8)	90.8 (± 35.9)	133 (± 50.1)	112.6 (± 10.9)	0.7 (± 0)	89.4 (± 8.9)	5.7 (± 0.4)	5.1 (± 1.8)
3	4	35.1 (± 5.9)	186.2 (± 30.9)	104.8 (± 33.6)	120.5 (± 53.1)	109.5 (± 15)	0.7 (± 0.1)	92.3 (± 16.6)	5.4 (± 0.5)	3 (± 1.5)
6	1	35.7 (± 6.4)	205.7 (± 35.7)	128.5 (± 34.9)	97.5 (± 35.4)	114.9 (± 15.2)	0.7 (± 0.1)	94.1 (± 12.6)	5.5 (± 0.3)	2.8 (± 1.5)
6	2	34.3 (± 5.1)	189.5 (± 28.5)	114.3 (± 28.8)	107 (± 20.7)	111.6 (± 13.6)	0.7 (± 0.1)	86.2 (± 7.1)	5.5 (± 0.4)	3.5 (± 2.5)
6	3	38 (± 8.8)	184.2 (± 29.9)	114.7 (± 28.8)	98.6 (± 30.1)	108.7 (± 16.7)	0.7 (± 0.1)	89 (± 7.4)	5.5 (± 0.4)	3.6 (± 1.4)
6	4	35.3 (± 4)	191.2 (± 30)	117.2 (± 18.5)	82.3 (± 24.9)	107 (± 13.6)	0.6 (± 0.1)	91 (± 16.9)	5.4 (± 0.3)	3.9 (± 2.3)
12	1	35.8 (± 5.3)	210.2 (± 27.5)	138.5 (± 28.2)	109.1 (± 43.2)	113.4 (± 10.5)	0.7 (± 0.1)	95.6 (± 15.8)	5.5 (± 0.4)	1.9 (± 0.8)
12	2	36.6 (± 5.3)	185.3 (± 41)	98.5 (± 26.9)	117.8 (± 46.1)	121.6 (± 16.4)	0.7 (± 0)	81.4 (± 8.3)	5.6 (± 0.3)	3.9 (± 2.5)
12	3	34 (± 4)	169.3 (± 12.7)	101.9 (± 12.2)	98 (± 28.8)	113.2 (± 9.1)	0.7 (± 0)	87 (± 7.9)	5.7 (± 0.3)	2.7 (± 1.4)
12	4	35.4 (± 8.6)	177 (± 21.7)	101.3 (± 17)	96.3 (± 77.9)	109.8 (± 20.7)	0.7 (± 0.1)	88.7 (± 9.9)	5.4 (± 0.4)	2.1 (± 0.9)

Data are expressed as mean ± SD Group 1 Mediterranean Diet (MD); Group 2 Liraglutide; Group 3 Ketogenic Diet (KD); Group 4 Metformin

*BMI* body mass index, *LDL* low-density lipoprotein, *TG* triglycerides, *Crfwaist* waist circumference, *CfrHratio* waist-to-height ratio, *FG* fasting glucose, *HbA1*_*c*_ Glycated Hemoglobin, *HOMA-IR* Homeostatic Model Assessment of Insulin Resistance

In the table (Table 3) shown below, we also aim to describe how the percentage of patients in each treatment group and at each specific follow-up time varied throughout the entire study period.

**Table 3:** Percentages of individuals remaining per treatment group relative to baseline

Months (from 0)	Group 1 (%)	Group 2 (%)	Group 3 (%)	Group 4 (%)	
3	40.6	88.5	100		73.7
6	18.8	46.2	100		36.8
12	12	53.8	44.4		26.3

*Group 1* Mediterranean Diet (MD); *Group 2* Liraglutide; *Group 3* Ketogenic Diet (KD); *Group 4* Metformin

### Analysis and graphical representation of variables

Regarding the analysis of BMI variations by therapy group, paired t-tests were conducted.

**Fig. 1:**
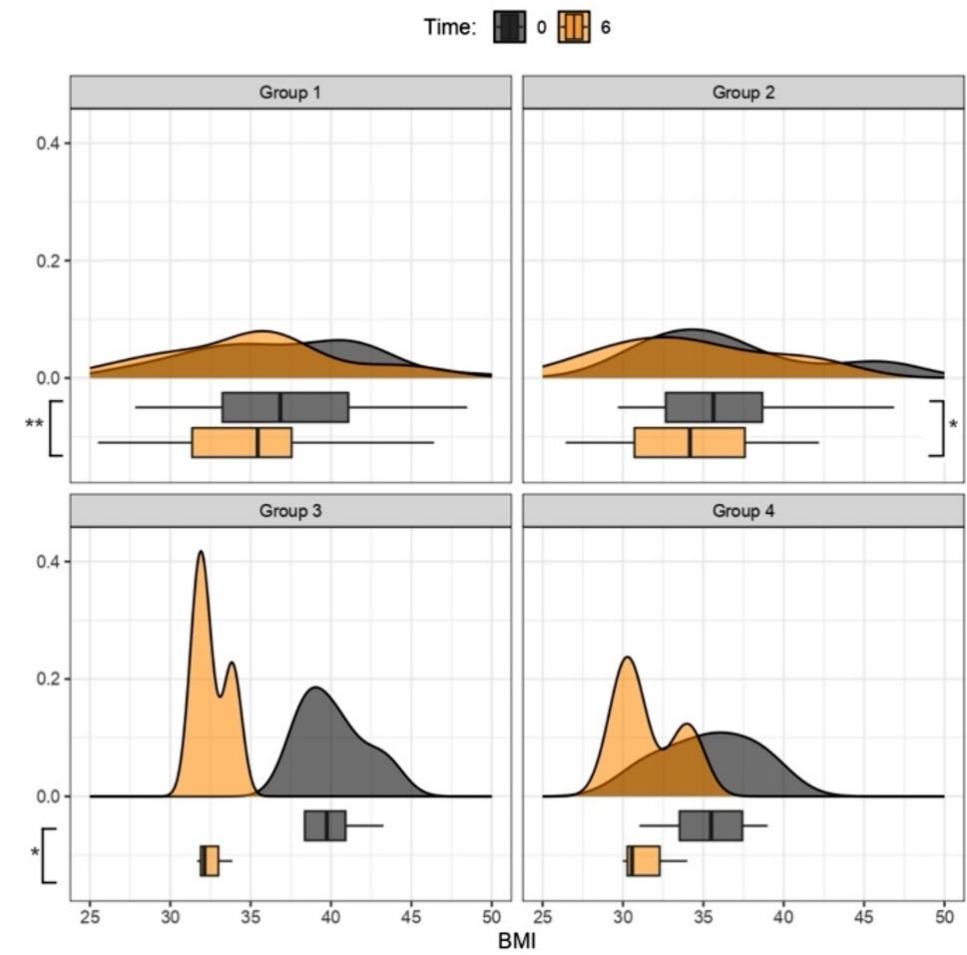
BMI (kg/m^2^) Variation between Time 0 and 6 months. Group 1: Mediterranean Diet; Group 2: Liraglutide; Group 3: Ketogenic Diet; Group 4: Metformin. The density curves above, accompanied by the box plots below, show a leftward shift, indicating a decrease in BMI scores across all treatment groups from 0 (grey graphs) to 6 months (orange graphs); p values < 0.05 (t-test for paired data) for Groups 1**, 2*, 3*, and *p* > 0.05 for Group 4

**Fig. 2:**
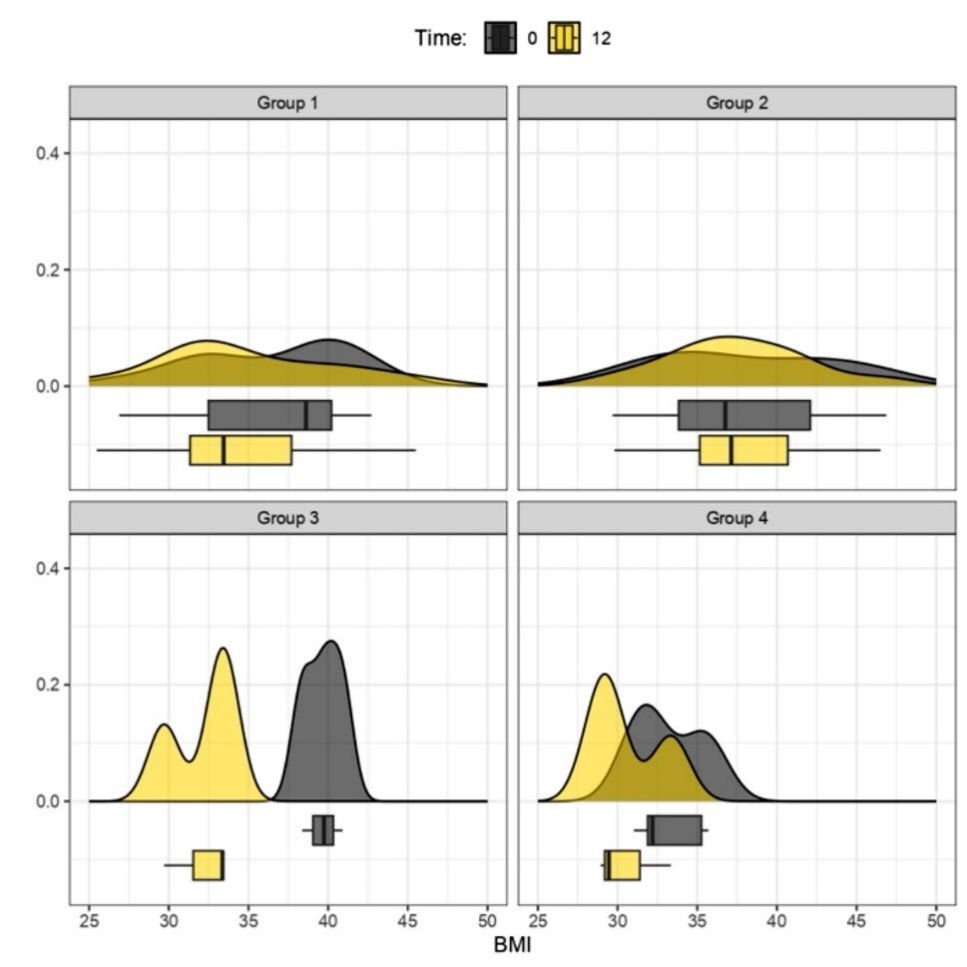
BMI (kg/m^2^) Variation between Time 0 and 12 months. Group 1: Mediterranean Diet; Group 2: Liraglutide; Group 3: Ketogenic Diet; Group 4: Metformin. The density curves above, accompanied by the box plots below, show a leftward shift for groups 1, 3, 4, indicating a decrease in BMI scores within their respective treatment groups from 0 (grey graphs) to 12 months (yellow graphs). Group 2 exhibits a steady trend between baseline (grey graphs) and 12 months (yellow graphs); p values > 0.05 (t-test for paired data) for Groups 1, 2, 3, 4

Figure 1 illustrates the overall reduction in BMI across the four therapeutic strategies during the initial 6 months, with a follow-up at 12 months (Fig. 2), involving the analysis of four distinct therapy groups. Graphs, represented by density curves accompanied at the bottom by box plots, were obtained for a more complete view of trends and medians for each therapy group.

Analysis of BMI by therapy group after 6 and 12 months of treatment (observation of the box plot at the bottom indicates BMI point loss):

Comparison at 12 months was further examined, specifically for all patients who initiated and completed a single treatment. In this case, as evidenced exclusively by the descriptive trends in the graphs, treatment 3 (KD) showed the highest BMI loss at 1 year. Moderate weight loss was observed for treatment 4 (metformin), and no significant BMI variations were evident for treatment 1 (MD).

Analysis of waist circumference and waist-to-height ratio variation after 3 and 6 months of treatment:

**Fig. 3:**
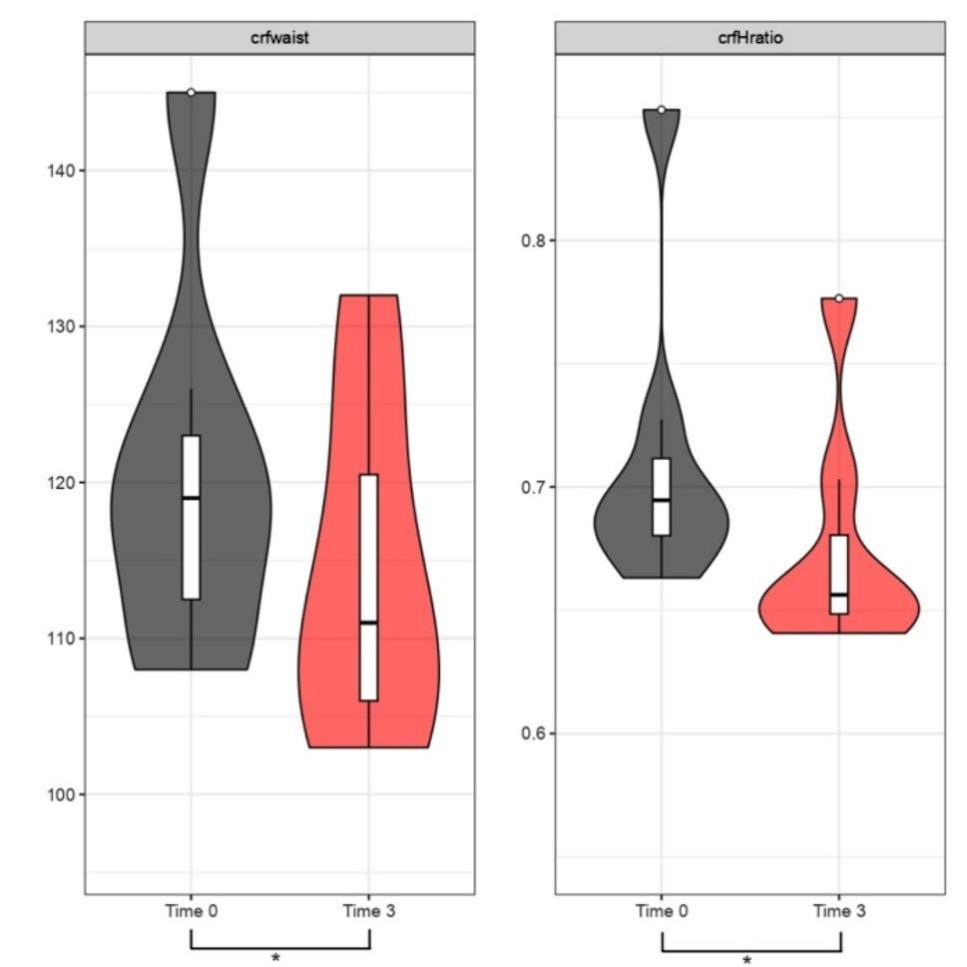
Waist Circumference (cm) and Waist-to-Height Ratio between Time 0 and 3 months. Specific analysis for Group 3 undergoing KD treatment. The violin plots in the first figure illustrate the trend of *crfwaist* between time 0 (grey plot) and after 3 months of treatment (red plot), showing a tendency towards downward movement, indicating a decrease in length in cm. A similar trend is observed for the *cfrHratio* value, with the red plot after 3 months of treatment indicating improvement, reflecting a downward shift; p-value (t-test for paired data) for *crfwaist* in Group 3 *p* < 0.05 (*) and for *crfHratio* Group 3 *p* < 0.05 (*)

**Fig. 4:**
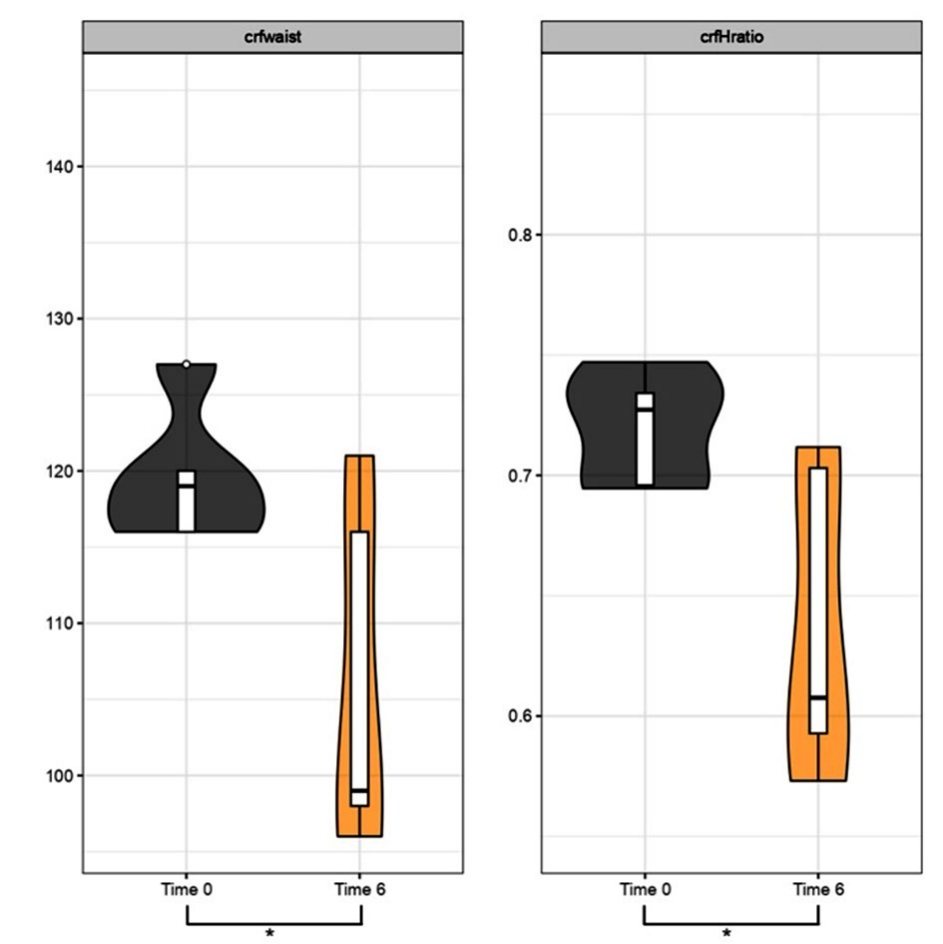
Waist Circumference (cm) and Waist-to-Height Ratio between Time 0 and 6 months. Specific analysis for Group 3 undergoing KD treatment. The violin plots in the first figure show the trend of *crfwaist* between time 0 (grey plot) and after 6 months of treatment (orange plot), with a tendency towards downward movement, indicating a decrease in length in cm. A similar trend is observed for the *cfrHratio* value, with the orange plot after 6 months of treatment showing improvement, indicating a downward shift; p value (t-test for paired data) for *crfwaist* Group 3 *p* < 0.05 (*) and for *crfHratio* Group 3 *p* < 0.05 (*)

### Analysis of Lipid Profile

**Fig. 5:**
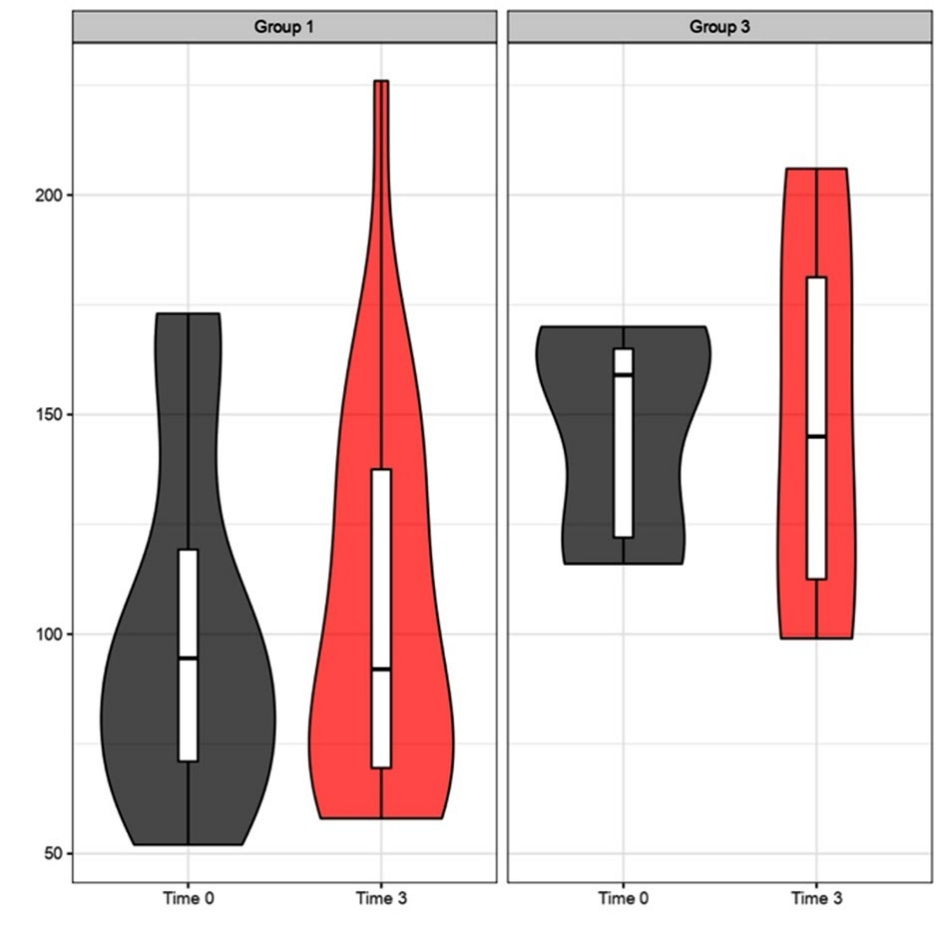
Triglyceride levels (mg/dL) after 3 months of treatment. Comparative analysis between Group 1 (MD) and Group 3 (KD). The violin plots at time 0 (grey plots) compared to 3 months (red plots) do not reveal a significant improvement trend for Group 1. Instead, for Group 3, a trend of improvement is noticeable in the right red plot, represented by the downward shift (decrease in triglycerides) of the ketogenic diet treatment group (on the right side); p values > 0.05 (t-test for paired data) for Groups 1, 3

**Fig. 6:**
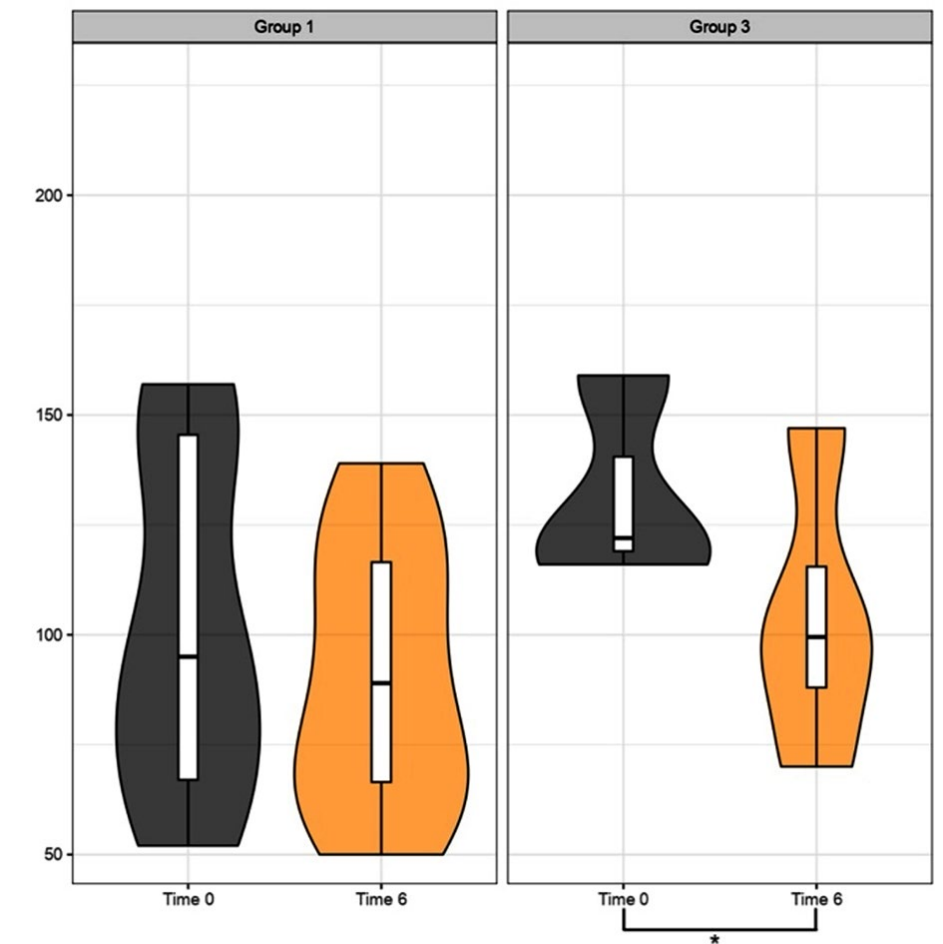
Triglyceride levels (mg/dL) after 6 months of treatment. Comparative analysis between Group 1 (MD) and Group 3 (KD). The violin plots at time 0 (grey plots) compared to 6 months (orange plots) do not reveal a significant improvement trend for Group 1. However, for Group 3, an improvement trend is observed in the right orange plot, represented by the downward shift of the treatment group’s median value; p values > 0.05 (t-test for paired data) for Groups 1 and p < 0.05 (*) for Group 3

Triglyceride levels after 3 and 6 months of treatment: the figures illustrate the trend in TG levels between Group 1 (MD) and Group 3 (KD) (Figs. 5 and 6).

**Fig. 7:**
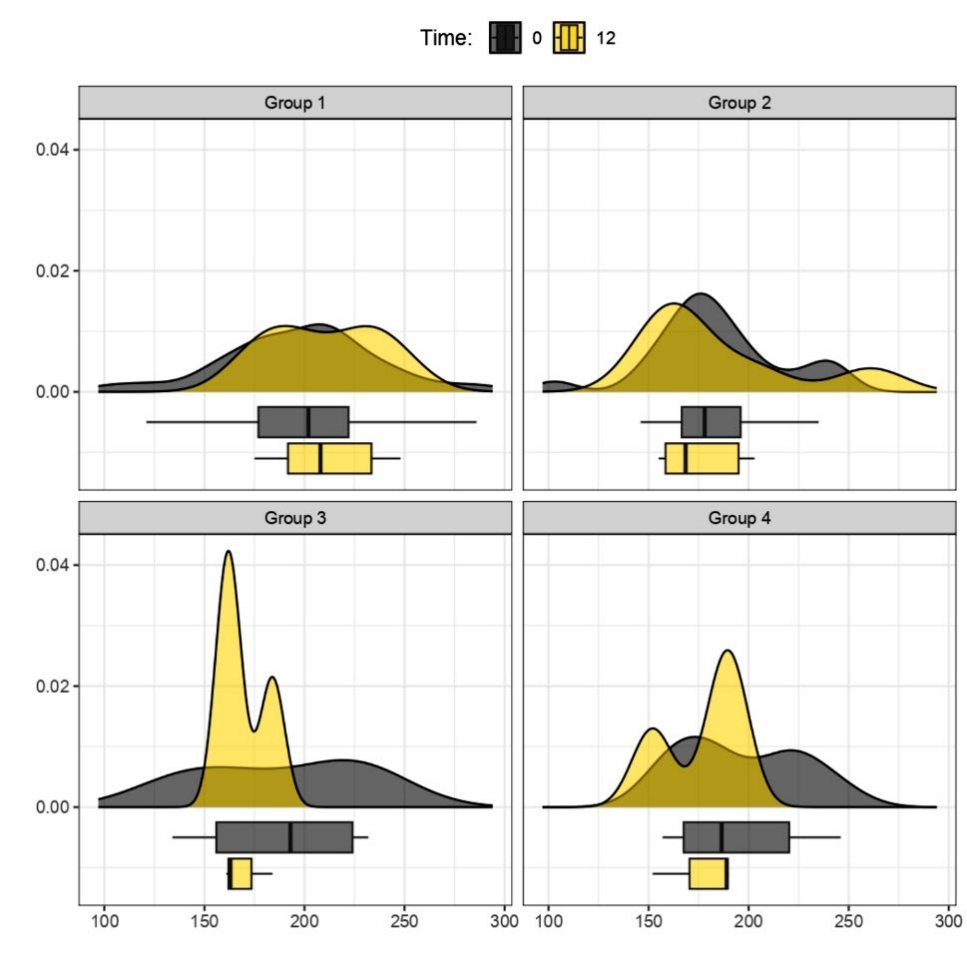
Total Cholesterol levels (mg/dL), comparison between Time 0 and 12 months. Group 1: Mediterranean Diet; Group 2: Liraglutide; Group 3: Ketogenic Diet; Group 4: Metformin. The graphs depicted by the density curves above and the box plots below aim to describe the trend of total cholesterol levels between time 0 (grey graphs) and 12 months (yellow graphs). There is no significant change in values overall, but especially the yellow graphs for Group 2 and Group 3 still show a tendency towards leftward movement (decrease in total cholesterol values); p values > 0.05 (t-test for paired data) for Groups 1, 2, 3, 4

**Fig. 8:**
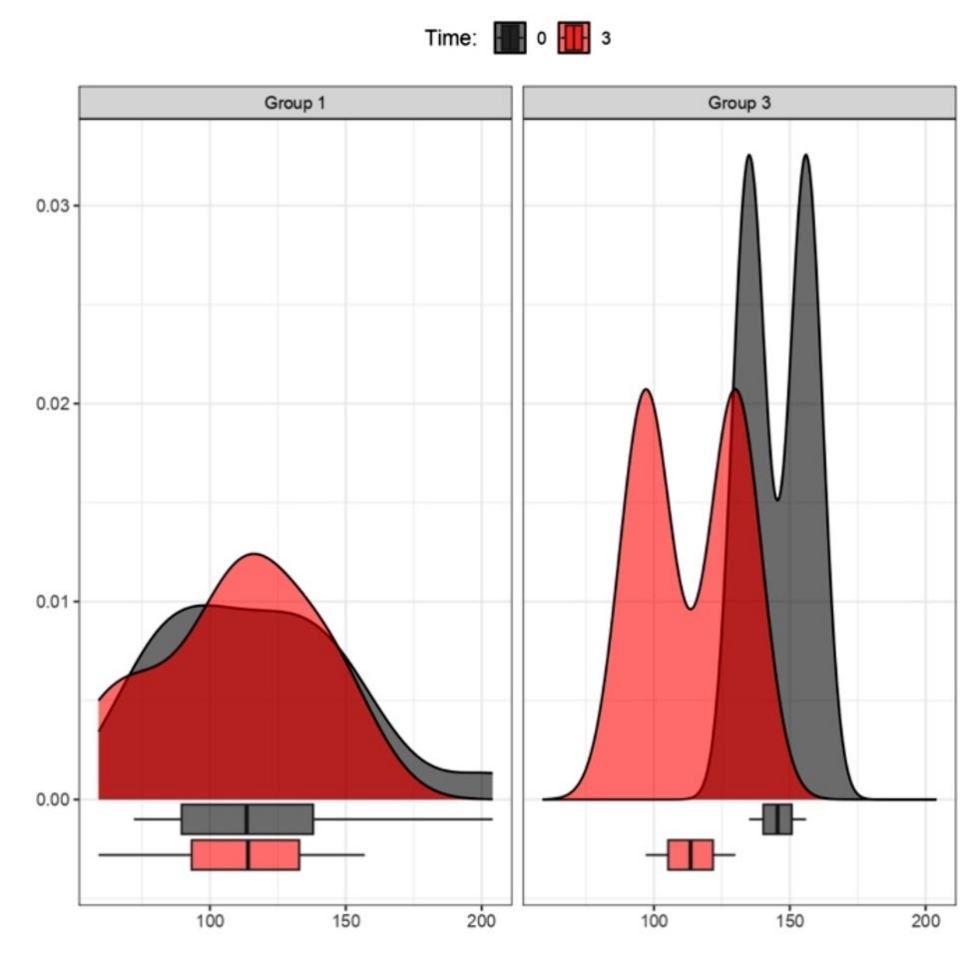
Comparison of LDL levels (mg/dL). Effect of MD (Group 1) and KD (Group 3) at Time 0 and after 3 months The graphs represented by the density curves above and the box plots below aim to describe the trend of LDL levels between time 0 (grey graphs) and 3 months (red graphs); no variation is shown for Group 1. For Group 3 on the right, the red curve at 3 months shows a tendency towards leftward movement (decrease in LDL values); p values > 0.05 (t-test for paired data) for Groups 1, 3

Regarding the analysis of the total cholesterol and LDL trends, the levels of total cholesterol are depicted in the comparison between 0 and 12 months for 4 therapy groups (Fig. 7), and the levels of LDL between 0 and 3 months are compared between the MD group and the KD group (Fig. 8).

For the analysis of the glycemic profile, we describe the results related to fasting glucose, followed by glycated hemoglobin (HbA1_c_), and finally, the HOMA Index.

**Fig. 9:**
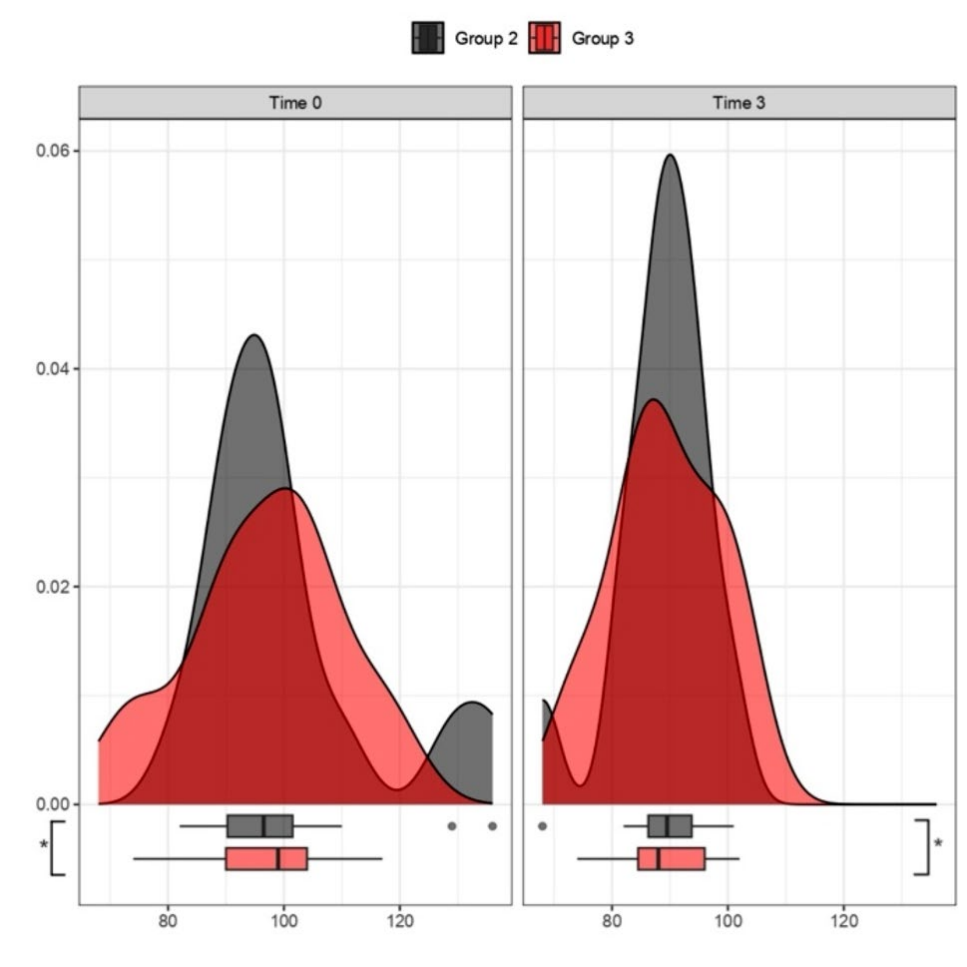
Analysis of fasting blood glucose levels (mg/dL). Effect of Liraglutide (Group 2) and KD (Group 3) at Time 0 and after 3 months. The graphs represented by the density curves above and the box plots below aim to describe the trend of FG levels between Group 2 (grey graphs) and Group 3 (red graphs). For both treatment groups in the right image (after 3 months), there is a trend of improvement in fasting glucose values, with a leftward shift (decrease in fasting glucose values); p values < 0.05 (t-test for paired data) for Groups 2 (*), 3 (*)

**Fig. 10:**
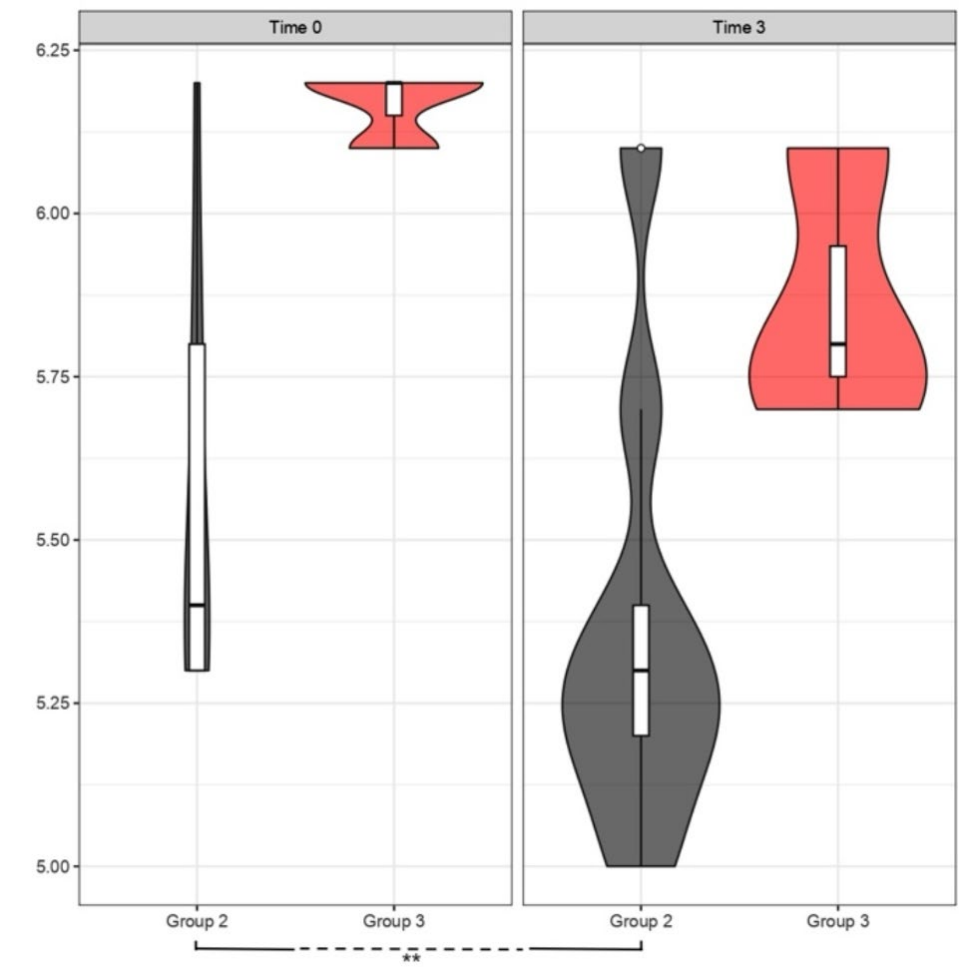
Comparison of HbA1_c_levels (%). Effect of Liraglutide (Group 2) and KD (Group 3) at Time 0 and after 3 months. The violin plots aim to describe the trend of HbA1_c_ levels between Group 2 (grey plots) and Group 3 (red plots). For both treatment groups, there is a trend of improvement in HbA1_c_ values, with a significant downward shift of both violin plots on the right (after 3 months of treatment) compared to the baseline on the left, highlighting a decrease in values; p value < 0.05 (t-test for paired data) for Group 2 (**), Group 3 *p* > 0.05

**Fig. 11:**
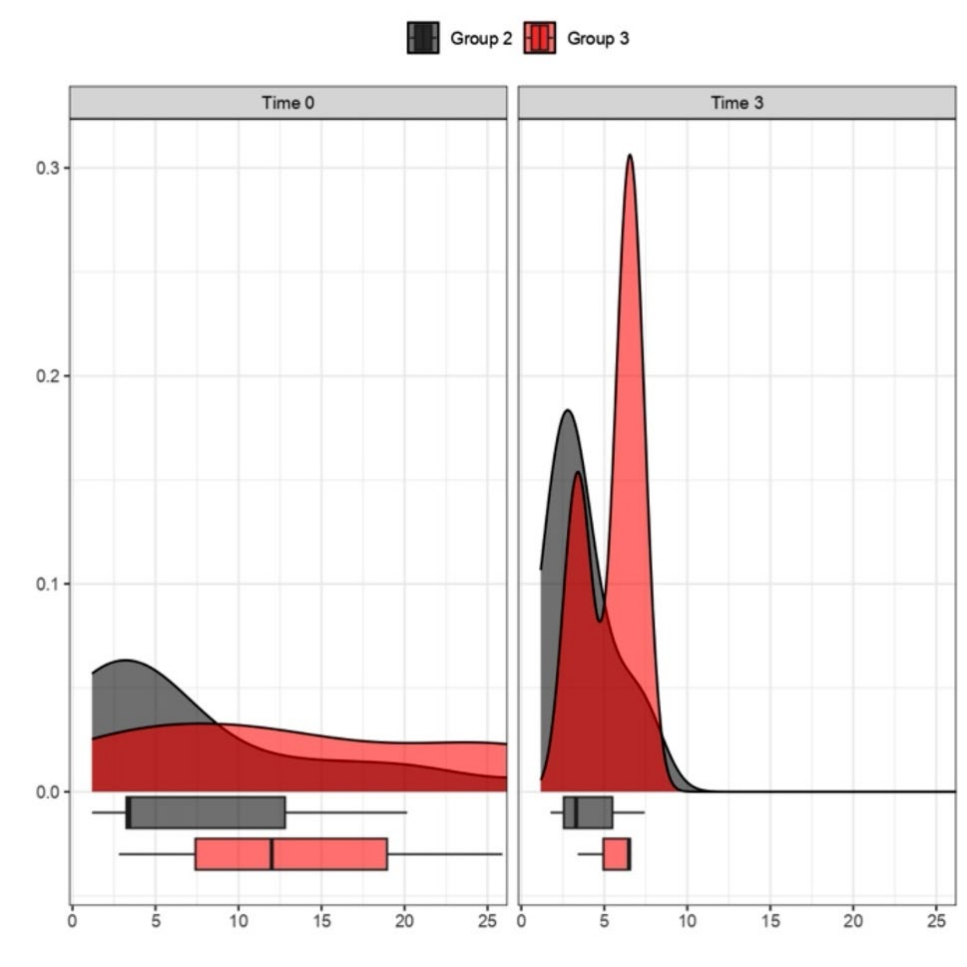
Comparison of HOMA-IR. Effect of Liraglutide (Group 2) and KD (Group 3) at Time 0 and after 3 months. The graphs represented by the density curves above and the box plots below aim to describe the trend of HOMA-IR levels between Group 2 (grey plots) and Group 3 (red plots). It is observed for both treatment groups in the right image a trend of improvement in HOMA-IR values, with a leftward shift, highlighting a decrease in values; p values > 0.05 (t-test for paired data) for Groups 2, 3

In the following figure (Fig. 10), the comparison of glycated hemoglobin trends between 0 and 3 months is highlighted through a violin plot, specifically comparing Group 2 (Liraglutide) and Group 3 (KD). Lastly, as the final analysis to study insulin resistance levels, we utilized the HOMA-IR; the figure (Fig. 11) illustrates the variation between time 0 and time 3 months in the therapeutic comparison between Liraglutide (Therapy 2) and KD (Therapy 3). Both treatments show a decrease in HOMA-IR in the first 3 months of treatment: the density curves at 3 months specifically highlight this trend, with a shift to the left.

## Discussion

As deduced from the results, each therapeutic strategy presents distinct advantages, which become valuable in relation to the individual patient profile and the appropriate timing of the specific therapy. In the following paragraphs, the evidence related to specific categories of variables will be analyzed in detail, starting with the analysis of anthropometric data, and then proceeding to the examination of results related to the lipid and glycemic profiles.

### Analysis of BMI, waist circumference, and waist-to-height ratio

The results have shown that during the first 6 months of treatment, Groups 1 (MD) – 2 (liraglutide) – 3 (KD) demonstrated a statistically significant reduction in BMI, with a p-value < 0.05. As in other studies, our work also highlighted that the VLCKD protocol was well-tolerated, with only mild and transient side effects. It demonstrated significantly greater effectiveness compared to a conventional diet with moderate and controlled carbohydrate intake [27]. In addition to BMI, we evaluated waist circumference and waist-to-height ratio as indicators of abdominal adiposity. The data showed statistical significance in the reduction of both variables in Group 3 (KD), with a p-value < 0.05, at 3 as well as 6 months. Nevertheless, it is crucial to highlight that, for the most part, individuals within Group 2 (Liraglutide) faced challenges in obtaining the medication due to a worldwide shortage. The shortage influenced treatment adherence, often resulting in intermediate dosages instead of maximum dosages or even treatment interruptions (the most used intermediate dosage was 1.8 mg/day). In this context, we also aim to briefly reflect on the use of metformin, a well-established drug in the field of diabetology, worthy of further exploration for the treatment of obesity. We emphasize that its integration into obesity clinics, irrespective of diabetes management, is easily achievable. Metformin represents a cost-effective option with minimal side effects that exert negligible impact on the quality of life. Our clinical experience suggests that patients, particularly those in the younger demographic, generally embrace a medication with these characteristics, especially when administered orally. The impact of metformin on reducing body weight, improving metabolism, and enhancing insulin responsiveness has secondary implications for reducing systemic inflammation. In scientific literature, it is now recognized that the effectiveness of metformin in reducing cardiovascular events has been demonstrated through controlled studies, although the therapeutic mechanisms of this drug are not entirely known and clarified [28, 29].

### Lipid profile

We conducted a thorough analysis of the lipid profile of patients, focusing specifically on triglycerides, total cholesterol, and LDL cholesterol. The data revealed a significant decrease in triglyceride levels in Group 3 (KD) with a p-value < 0.05 after 6 months, showing a similar trend to what was observed for Body Mass Index (BMI), as supported by the literature [30]. Furthermore, Group 3 (KD) exhibited a significantly greater impact on both total cholesterol and, solely in the short term, LDL cholesterol compared to the other treatment groups. For LDL cholesterol, we presented data from the acute phase to highlight the benefits derived from our observations during the initial stage of the VLCKD. It is crucial to remember that among the benefits of the Ketogenic Diet, the reduction of LDL is not included [31]. However, we want to emphasize that, in any case, we should focus not only on LDL cholesterol levels per se but also on the composition of lipoproteins, which more accurately determines the risk [31–34]. Furthermore, there is a correlation between the dimensions of LDL particles and variables such as obesity and insulin resistance [31, 35].

### Glycemic profile

For the analysis of the glycemic profile, we focused our attention on fasting blood glucose. In the first three months of treatment, both Group 2 (Liraglutide) and Group 3 (KD) demonstrated a significant reduction, with a p-value < 0.05. The benefits of treatment with drugs belonging to the GLP1-RA class in relation to the reduction of fasting glucose levels and glycated hemoglobin have been extensively documented [36], a reality reaffirmed in our case studies where Liraglutide was used with the primary goal of promoting weight loss. However, it is crucial to emphasize that Group 2 (liraglutide) showed a statistically significant decrease in HbA1_c_ with a p-value < 0.05 at three months. In this situation, it is essential to consider the previous observations regarding GLP1-RA treatment and the factors influencing the overall effectiveness of long-term treatment. Additionally, another noteworthy result emerged from the Analysis of Variance (ANOVA) conducted between Group 2 and Group 3 in relation to the time variable. This analysis revealed that, during the first three months, the time factor exerts a greater influence than the direct effects of the treatments themselves on the variation in fasting blood glucose levels (*p* < 0.05). This leads us to posit the concept that, presumably, in patients with obesity, adherence to treatment in the initial three months of therapy is likely more crucial than the type of treatment itself. As for glycated hemoglobin (HbA1_c_), it should be noted that in Group 2 (Liraglutide), a significant decrease was observed between the start of treatment and the end of the three months, with *p* < 0.05. Furthermore, it is relevant to note that both treatments (KD and Liraglutide) contributed to reducing HOMA-IR in the first three months, as clearly evidenced by density curves. Regarding the VLCKD, the well-established benefit on insulin resistance levels and glycated hemoglobin has been confirmed for some time [30]. Therefore, we can employ ketosis through Ketogenic Diet protocols, mindful that we are simultaneously reducing both glucotoxicity and lipotoxicity, which are foundational to adipose tissue-related diseases. All of this is rooted in the attenuation of hyperinsulinemia and the metabolic shift towards lipid oxidation, utilizing fatty acids and ketones for energy [37, 38].

## Conclusion

This study presents the results obtained at our dedicated obesity clinic during a very particular period in which we had to face shortages of medications. The work aims to emphasize that the proposed medical and dietary strategies are not mutually exclusive but can be integrated to optimize outcomes. To our knowledge, there are currently no recognized literature studies with established protocols for alternating therapeutic approaches between ketogenic diets and pharmacological obesity treatment. We have shared our real-life experience with the aim of contributing to the dissemination of straightforward and effective therapeutic strategies. We chose graphical representation to appreciate the trends of the examined parameters in relation to follow-up timelines, carefully selecting parameters widely used in clinical practice for metabolic assessments. Therapeutic decisions are particularly complex as they require a highly personalized approach. The management of obesity requires a holistic approach that involves a varied group of healthcare professionals, including specialized physicians, dietitians, psychologists, and exercise science experts, to provide comprehensive, multidisciplinary care. Additionally, it is crucial to consider that obesity treatment cannot be limited to short-term solutions but requires strategic planning in the medium and long term. Obesity is a chronic and recurrent condition; therefore, its treatment must be equally chronic and highly personalized.

## Data Availability

The datasets employed or analyzed in the present study are accessible upon reasonable request from the corresponding author.

## Abbreviations

KD: Ketogenic Diet
MD: Mediterranean Diet
F: Female
M: Male
HOMA-IR: Homeostatic Model Assessment of Insulin Resistance
HbA1_c_: Glycated Hemoglobin
ANOVA: Analysis of Variance
TG: Triglycerides
BMI: Body Mass Index
LDL: Low-Density Lipoprotein
Cfrwaist: Waist Circumference
CfrHratio: Waist-to-height ratio
FG: Fasting Glucose
FDA: Food and Drug Administration
EMA: European Medicines Agency
GLP1-RA: Glucagon-Like Peptide-1 Receptor Agonists
VLCKD: Very Low-Calorie Ketogenic Diet
SD: Standard Deviation
